# AT2R -1332 G:A polymorphism and diabetic nephropathy in type 2 diabetes mellitus patients

**DOI:** 10.12861/jrip.2013.31

**Published:** 2013-09-01

**Authors:** Zohreh Rahimi, Omid Mansouri Zaveleh, Ziba Rahimi, Ardeshir Abbasi

**Affiliations:** ^1^Medical Biology Research Center, Kermanshah University of Medical Sciences, Kermanshah, Iran; ^2^Department of Biochemistry, Medical School, Kermanshah University of Medical Sciences, Kermanshah, Iran; ^3^Department of Biochemistry, Sanandaj Science and Research Branch, Islamic Azad University, Sanandaj, Iran

**Keywords:** Type 2 diabetes mellitus, AT2R -1332 G:A, Diabetic nephropathy, Polymorphism

## Abstract

**Introduction:** The rennin-angiotensin system (RAS) plays a central role in the regulation of sodium metabolism, vascular tone, blood pressure, renal hemodynamics, and vascular modeling and is activated by hyperglycemiaObjectives: In the present study the influence of AT2R -1332 G:A polymorphism on the risk of T2DM and its complications in a population from Western Iran has been investigated.

**Patients and Methods:** In a case-control study, 70 individuals with type 2 diabetes mellitus (T2DM) including normo-, micro- and macro-albuminuric patients and 112 healthy subjects from the Kermanshah province were studied to investigate the association between the angiotensin type 2 receptor (AT2R) -1332 G:A variants with the risk of T2DM and its complications. The genotypes of the AT2R were detected using polymerase chain reaction-restriction fragment length polymorphism (PCR-RFLP) method. Analysis of AT2R -1332 G:A polymorphism indicated the absence of association between this polymorphism with T2DM and diabetic nephropathy.

** Results:** Analysis of AT2R -1332 G:A polymorphism indicated the absence of association between this polymorphism with T2DM and diabetic nephropathy. In females with diabetic nephropathy a significantly higher frequency of AA genotype (50%) was detected compared to those without nephropathy (13.3%, p=0.015). The presence of A allele of AT2R was associated with significantly (p=0.029) increased risk of coronary artery disease (CAD) in diabetic patients without nephropathy.

** Conclusion:** Our study indicated an association between the AT2R -1332 G:A polymorphism and the risk of diabetic nephropathy in females only. Also, the A allele was associated with the risk of CAD in those diabetic patients without nephropathy.

**Implication for health policy/practice/research/medical education:**

In a case-control study on 70 individuals with type 2 diabetes mellitus, we found that the presence of A allele of AT2R is associated with the risk of coronary artery disease in diabetic patients without nephropathy.

## Introduction

The rennin-angiotensin system (RAS) plays a central role in the regulation of sodium metabolism, vascular tone, blood pressure, renal hemodynamics, and vascular modeling and is activated by hyperglycemia (1). In diabetic patients hyperglycemia increases tissue angiotensin II which induces oxidative stress, glomerular hyperfiltration, endothelial damage, thrombosis, inflammation and vascular remodeling (2). Angiotensin II binds to two main types of receptors. The angiotensin type 1 receptor (AT1R) mediates vasoconstriction and the proliferative action of angiotensin II, while the type 2 receptor (AT2R) inhibits cell proliferation and mediates apoptosis and works as a cardio protective agent against AT1R (1,3). Diabetic nephropathy (DN) starts with various renal functional changes including glomerular hyperfiltration and hyperperfusion, and is manifested with microalbuminuria that subsequently can progress to macroalbuminuria (4). Diabetic nephropathy and end-stage renal diseases are major causes of mortality in diabetes mellitus (1,5).

The AT2R gene is located on the chromosome X at the locus Xq23–26. The AT2R gene consists of three exons and two introns. A common AT2R polymorphism is located within intron 1, 29 bp before the start of exon 2, close to a region that is important for transcriptional activity. This polymorphism is designated as -1332 G:A according to the translation initiation site of the gene, although it has also been described as +1675 G:A (6).

The G allele of AT2R has already been associated with congenital anomalies of the kidney and urinary tract in men (7).

However, the literature does not contain any information on the influence of AT2R -1332 G:A variants in the development of the risk of type 2 diabetes mellitus (T2DM) and its complications.

## Objectives

In the present study the influence of AT2R -1332 G:A polymorphism on the risk of T2DM and its complications in a population from Western Iran has been investigated.

## Materials and Methods

### Sample

AT2R -1332 G:A genotypes were studied in 70 T2DM patients including 28 patients with micro-, 22 with macro- and 20 with normo-albumiuria and 112 healthy subjects.

The patients had been admitted to the Taleghani Diabetes Research Center of Kermanshah University of Medical Sciences and all were from Kermanshah Province of Iran with Kurdish ethnic backgrounds. Type 2 diabetes mellitus was diagnosed according to WHO criteria (8).

The criteria for defining microalbuminuria and macroalbuminuria were albumin to creatinine ratio, (ACR) of 30–299 mg/g and ≥300 mg/g, respectively in a random spot collection of urine in three specimens collected within a 3–6 months period. To confirm the presence of micro- or macro-albuminuria in samples with ACR’s higher than 30 mg/g, ACR was measured in 24 h urine collection. Diabetic patients with ACR <30 mg/g made up the normo-albuminuric patients (9).

Detailed medical history of each patient was provided. Informed written consent was obtained from each individual before participation. The study was approved by the Ethics Committee of Kermanshah University of Medical Sciences and was in accordance with the principles of the Declaration of Helsinki II.

### Genotype analysis

DNA was extracted from the leukocyte fraction of the EDTA-treated whole blood using the phenol-chloroform method (10).

The AT2R -1332 G:A polymorphism was genotyped using the primers of 5'-GGA AGG TAG AAC ATA CAT TAA ATG-3' and 5'-AGA GAA ACA GCA GCT AAA GAA TT-3'. The PCR product with 120-bp was digested with EcoRI restriction enzyme. In the presence of G allele two fragments with 91- and 29-bp fragments were produced, while in the presence of A allele the 120-bp fragment remained intact (11).

### Statistical analysis

The allelic frequencies were calculated by the chromosome counting method. The significance of differences in genotype and allele frequencies of AT2R -1332 G:A between patients and controls were calculated using χ^2^ test. Odds ratios (OR) were calculated as estimates of relative risk for disease and 95% confidence intervals (CI) were obtained by SPSS logistic regression software. A two-tailed Student's *t*-test was used to compare quantitative data. Statistical significance was assumed at p<0.05 level. The SPSS 16 (SPSS Inc., Chicago, IL, USA) was used for the statistical analysis.

## Results

The demographic and biochemical characteristics of diabetic patients are depicted in Table 1. The mean age of normo-, micro- and macro-albuminuric patients were 53.1 ±10.1, 52.4±6.5 and 53.9±9.3 years, respectively. Diastolic blood pressure was significantly higher in macroalbuminuric patients (87.3±7.2 mmHg, p=0.014) compared to normoalbuminuric ones (80.3±10.3 mmHg). The frequency of the AT2R alleles in patients and healthy individuals has been indicated in Table 2. As demonstrated in Table 2, the frequency of A allele in all diabetic patients was non significantly higher (34.3%, p=0.74) than that in healthy individuals (32.1%).

**Table 1:** Characteristics of diabetic patients.

**Variables**	**Normoalbuminric patients** ** (n=20)**	**Microalbuminuric patients (n=28)**	**Macroalbuminuric patients** ** (n=22)**
Age (years)	53.1±10.1	52.4±6.5 p=0.76	53.9±9.3 p=0.79
BMI (Kg/m^2^)	27.0±4.3	27.5±4.2 p=0.68	26.9±4.1 p=0.95
HbA_1C_ (%)	7.7 ±1.3	8.0 ±1.76 p=0.58	8.1 ±2.1 p=0.54
Diabetes duration (years)	8.6±6.2	8.0±5.1 p=0.73	10.8±6.0 p=0.24
Systolic blood pressure (mmHg)	127.8±19	129.5±20.7 p=0.77	137.5±20.3 p=0.11
Diastolic blood pressure (mmHg)	80.3±10.3	81.1±10.9 p=0.78	87.3±7.2 p=0.014
Triglycerides (mg/dl)	148.7±62.6	146.2±46.1 p=0.87	156.3±65.5 p=0.7
Cholesterol (mg/dl)	180.7±25.9	175±36 p=0.55	165.4±38.9 p=0.14
LDL-C (mg/dl)	101±22.2	89.3±24.1 p=0.096	100.9±28.8 p=0.98
HDL-C (mg/dl)	41.7±9	45.8±16.2 p=0.31	44.6±22.5 p=0.59
*Comparison has been made with normoalbuminuric patients.			

**Table 2:** The comparison of frequency of AT2R alleles between diabetic patients and the healthy controls.

	Diabetic patients without nephropathy (n=40)	Diabetic patients with nephropathy (n=100)	All diabetic patients (n=140)	Healthy subjects (n=224)
AT2R alleles				
G	23 (57.5%)	69 (69%)	92 (65.7%)	152 (67.9%)
A	17 (42.5%)	31 (31%)	48 (34.3%)	72 (32.1%)
	(χ^2^=1.89, df=1, p=0.16)*		(χ^2^=0.1, df=1,p=0.74)**	
*Comparison has been made with diabetic patients with nephropathy. ** Comparison has been made with healthy subjects.				

The frequencies of AT2R -1332 G:A genotypes and alleles in females and the frequency of alleles in hemizygous males are demonstrated in Table 3. A significantly higher frequency of AA genotype (50%, p=0.016) was observed in females with nephropathy compared to those without nephropathy (13.3%). However, no significant difference was detected between patients with and without nephropathy and also between patients with healthy individuals related to the frequency of AT2R genotypes and alleles.

**Table 3:** The distribution of AT2R -1332 G:A genotypes and alleles in diabetic females and males with and without nephropathy and healthy individuals.

	T2DM without Nephropathy (n=21)	T2DM with Nephropathy (n=49)	All Diabetic Patients (n=70)	Healthy Individuals (n=112)
Females	n=15	n=32	n=47	n=92
AT2R genotypes				
GG	5 (33.3%)	2 (6.2%)	7 (14.9%)	9 (9.8%)
GA	8 (53.3%)	14 (43.8%)	22 (46.8%)	48 (52.2%)
AA	2 (13.3%)	16 (50%)	18 (38.3%)	35 (38%)
(χ^2^=8.81, df=2, p=0.012)*		(χ^2^=0.96, df=2, p=0.61)**		
GG+GA	13 (86.7%)	16 (50%)	29 (61.7%)	57 (62%)
AA	2 (13.3%)	16 (50%)	18 (38.3%)	35 (38%)
(χ^2^=5.81, df=1, p=0.016)*		(χ^2^=0.006, df=1, p=0.93)**		
Males	n=6	n=17	n=23	n=20
AT2R alleles				
G	0 (0%)	6 (35.3%)	6 (26.1%)	3 (15%)
A	6 (100%)	11 (64.7%)	17 (73.9%)	17 (85%)
(χ^2^=2.86, df=1, p=0.091)*		(χ^2^=0.79, df=1, p=0.37)**		
*Compared to nephropathy patients ** Compared to healthy individuals				

In diabetic patients without nephropathy the A allele of AT2R was present in 60% of these patients with a history of coronary artery disease (CAD). However, in those patients without history of CAD the frequency of this allele reached to 25% (p=0.025). The presence of this allele was associated with a 4.5-fold (95%, CI= 1.16-1.37, p=0.029) increased risk of CAD in diabetic patients without nephropathy.

## Discussion

The anti-proliferative actions of the AT2R offset the growth promoting effects mediated by the AT1R. It has been suggested that reduced transcription of the AT2R gene in the presence of G allele might produce clinical effects resulting from a decreased production of AT2R. Experimental antagonism of AT2R results in an increase in systolic blood pressure. To the contrary, stimulation of the AT2R is associated with a vasodilator cascade involving increased production of bradykinin and nitric oxide (6). In a more complicated system with a combined hypertension and insulin resistance, insulin and RAS may cross-talk with each other. After blocking AT1R signaling pathway, unbound angiotensin II can act on AT2R. The stimulation of AT2R can regulate insulin sensitivity at multiple sites of the insulin signaling pathway, and also regulate vascular remodeling in concert with insulin (12). However further study is needed to clarify the direct cross-talk of insulin and angiotensin II mediated signaling through the AT1R and AT2R in glucose metabolism and vascular modeling. The AT2R G allele was reported to be associated with congenital anomalies of the kidney and urinary tract in a small study of males only (7).

The present study indicates a higher but not significantly different frequency of AT2R A allele in diabetic patients compared to controls. However, we observed an association between the risk of diabetic nephropathy with AA genotype of AT2R in female patients only. Herrmann *et al*. (13) investigated the AT2R gene -1332 G/A polymorphism and observed an increase in the frequency of the G allele in men with ischemic heart disease. However, they also reported increased frequency of the A allele in a subgroup of females with a history of ischemic heart disease. This finding was dependent on the inclusion of heterozygous females, despite the polymorphism being X-linked.

We found that the presence of A allele of AT2R is associated with the risk of CAD in diabetic patients without nephropathy. Unlike AT1R, the role of AT2R in controlling blood pressure and cardiovascular function has not yet been known, as the AT2R concentration in the adult cardiovascular system is low (12). Alfaikh **et al*. *(6) observed an association of AT2R -1332 G allele and premature CAD in hemizygous males. However, in two cohort studies, association of AT2R A allele with the elevated left ventricular mass in young men with mild hypertension (14) and in females with ischemic heart disease (13) have been demonstrated. Since, both the A and the G alleles of AT2R -1332 G:A have been independently associated with left ventricular remodeling, it needs to be confirmed at the molecular level to find out if the carriers of the G allele express more or less AT2R compared to the carriers of the A allele (15). Also, the potential mechanisms by which AT2R -1332 G:A polymorphism might be involved in the protection against microalbuminuria requires elucidation.

## Conclusion

In summary, our study indicated a non-significantly increased frequency of AT2R A allele in diabetic patients compared to controls. We observed an association between the risk of diabetic nephropathy with AA genotype of AT2R in female patients only. Also, we found that the presence of A allele of AT2R is associated with the risk of CAD in diabetic patients without nephropathy.

## Authors' Contributions

ZR defined the aims of research and prepared the final paper. Zi R, OMZ and AA performed experiments and prepared the manuscript.

## Conflict of interests

The authors declare that, they have no conflict of interest.

## Ethical considerations

Ethical issues (including plagiarism, data fabrication, double publication) have been completely observed by the author.

## Funding/Support

This work was financially supported by a grant from vice chancellor for research of Kermanshah University of Medical Sciences, Kermanshah, Iran.
